# Autumn Migration of Greater Noctule Bat (*Nyctalus Lasiopterus*): through Countries and over Mountains to a New Migration Flight Record in Bats

**DOI:** 10.1134/S0012496623700746

**Published:** 2023-11-11

**Authors:** D. A. Vasenkov, N. S. Vasiliev, N. V. Sidorchuk, V. V. Rozhnov

**Affiliations:** 1 Severtsov Institute of Ecology and Evolution, Russian Academy of Sciences, Moscow, Russia; 2 Moscow State University, Moscow, Russia

**Keywords:** greater noctule bat, *Nyctalus lasiopterus*, seasonal migration, GPS-GSM trackers

## Abstract

For the first time, using GPS-GSM trackers, long-term seasonal flights of greater noctule bat (*Nyctalus lasiopterus*) from summer habitats in Russia to wintering areas in Europe are recorded. One of the seasonal migratory flights is a record distance for bats (2515 km). The maximum daily flight was 445 km. One of the animals abruptly changed the direction of migration from southwest to north on the third day of flight after crossing the Kursk magnetic anomaly.

Seasonal migrations are an important stage in the life of many animal species. For some groups, such as birds, migration has been extensively studied for a long time. Due to such studies, it has been possible to answer many questions regarding animal ecology, participation in the transfer of matter and energy in ecosystems at different levels, and interaction with other organisms. In addition, it is important to study migratory species participation in transmission of potential human and animal pathogens [1]. Among terrestrial vertebrates, mass regular migrations are known not only for birds, but for bats also. However, the level of chiroptera migration information is much lower than for birds, for various reasons, including methodological difficulties caused by the small size of these mammals and their nocturnal activity. Due to long-term ringing of different bat species in Russia [2], Europe and North America, the approximate areas of their wintering and summer habitats have become known [3, 4]. Daily traced movement routes were obtained only for large species - fruit bats which move in search of fruit-bearing plants within tropical and subtropical regions of Africa and Australia [5, 6]. For “true” migratory bat species, similar seasonal migrations detailed studies have not been possible previously. Only for one of the largest Europe bat species—the common noctule *Nyctalus noctula*—just the initial stages of migration were studied [7].

The purpose of this study was to study the seasonal migration of the greater noctule bat (*Nyctalus lasiopterus*), the largest insectivorous species of bats in Europe, using GPS-GSM trackers.

## MATERIALS AND METHODS

To study the autumn migration of the greater noctule (*Nyctalus lasiopterus*), three individuals of this species were tagged with small-sized GPS-GSM trackers based on the MT2503 chip and aluminum rings with individual numbers. The animals were captured in their summer habitat in the Meshchera National Park in the vicinity of the village of Tikhonovo (Gus-Khrustalny District, Vladimir Region, Russia). Data on tagged individuals, the dates of their tagging and the start and end of tracker work are shown in Table 1.

**Table 1.** Periods of tracker operation and characteristics of tagged greater noctule bats

Tracker and ring	Sex and age	Date of			Tracking duration, days
		tagging (ringing)	migration start	end of the tracker work	
Tracker no. 51 (RUSSIA 55-00051)	Yearling female	September 12, 2020	September 19, 2020	October 4, 2020	22
Tracker no. 46 (RUSSIA 55-00046)	Adult female	September 18, 2021	September 28, 2021	October 11, 2021	23
Tracker no. 53 (RUSSIA 55-00053)	Yearling male	September 18, 2021	September 28, 2021	October 16, 2021	28

Age of yearling individuals is 3–4 months, age of an adult female is more than one year.

We previously presented the characteristics of GPS-GSM trackers [8]. To save battery power, the trackers are programmed to turn on briefly from a shake when the animal leaves the shelter. This allows us to register the daytime shelter coordinates. After turning on, the tracker transmits coordinates via the   cellular network to the website www.livegpstracks.com, and then turns off for 11 hours and goes into flight standby mode. If the tracker failed to register coordinates, its location was determined from the base stations of the cellular network which it contacted via tracking site http://www.livegpstracks.com. During migration, greater noctules moved at night, and usually spent daylight hours in a new shelter every day. Sometimes the animals remained in one shelter for more than one day, possibly due to weather conditions. We defined the migration start date as the night on which individuals moved more than 50 km from their summer habitat. The duration of trackers operation was limited by the battery power and probably did not last to the final point of the migratory flight. However, due to trackers work over approximately two weeks period, daily information on most of the migration routes for tagged *N. lasiopterus* individuals was obtained.

The tracking data were processed in the QGIS 3.10 program (www.qgis.org).

## RESULTS AND DISCUSSION

Greater noctule bats migration characteristics are presented in Table 2.

The data from GPS-GSM trackers showed three tracks with a duration of 13 to 18 days and a length of 1754 to 3360 km (Table 2). The straight-line distances between the start points of greater noctule migration and the end trackers data transmission points ranged from 1439 to 2515 km. The longest flight (both in track length and in straight line distance between the extreme points of migration) was made by male no. 53, and is currently the longest recorded migratory flight for bats.

**Table 2.** Flight parameters of three greater noctule bats during migration

Parameter	Female no. 51	Female no. 46	Male no. 53
Date of migration start	September 19, 2020	September 28, 2021	September 28, 2021
Duration of observed migration, days	15	13	18
Number of daily flights during migration, days	12	7	17
Number of stopovers during migration, days	3	6	1
Amount of “active” days* during migration, %	80	54	94
Migration length (in a straight line), km	1439	1678	2515
Length of the migration track, km	2135	1754	3360
Maximum daily flight, km	359	415	445
Minimum daily flight, km	14	64	49
Average daily flight, km	142	135	187
Average daily flight (excluding days of stopover), km	178	250	198

Days of stopover we defined as days during which no flights of the bats were recorded, or the flights were less than 3 km (previously, such a distance was repeatedly recorded by us, when the bats changed daytime roosts during the day); * by “active” days we mean the proportion (in %) of days with flights to the total number of days (with and without flights).

The maximum distance covered by the animals per night during autumn migration reached 445 km (male flight between first and second migration days), the minimum was 14 km (young female no. 51 flight at the end of its route recorded by the tracker). The average distance covered by the animals per day was 154.8 km, and excluding stops—208.7 km.

**Fig. 1.**
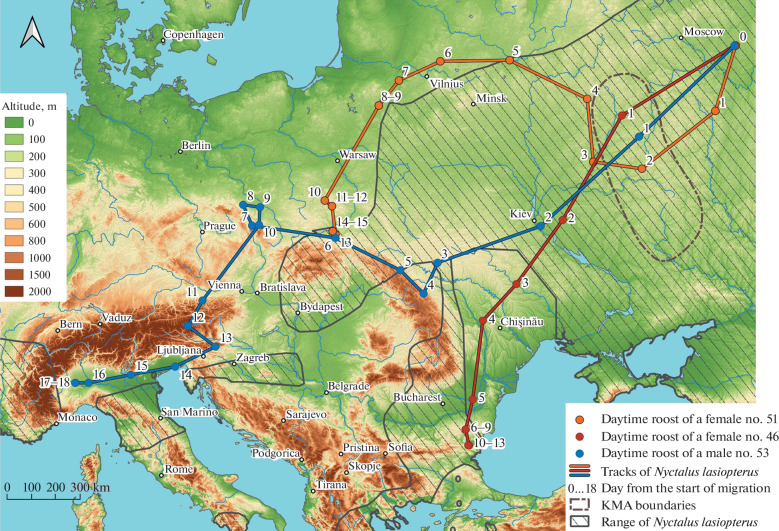
Autumn migration flights of three greater noctule bat tagged with GPS-GSM trackers. Numbers indicate the location of each individual, corresponding to the days of migration from its start date. The range of the greater noctule bat is given according to [9], the boundaries of the Kursk magnetic anomaly (KMA) according to [10].

The animals left their summer habitats in the second half of September: in 2020, young female no. 51 began the autumn migration flight on September 19, in 2021 two other animals (young male no. 53 and adult female no. 46) on September 29. The departure point of all three greater noctules is the vicinity of the village of Tikhonovo (Vladimir Region, Gus-Khrus-talny District), but their migration in the southwestern direction ran along different routes (Fig. 1).

The route of an adult female no. 46, which tracker stopped working in the east of Bulgaria, was the most “rational”: it is the shortest, the track length differs little from a straight line length between the start and end points of the route (1754 and 1678 km, ratio 1.05).

The route of young male no. 53 passed to the west, skirting the mountains in the middle part of the route, which is clearly visible on the orographic map. This tracker stopped working in northwestern Italy, about 100 km from the French border. This track is less rational (3360 and 2515 km, ratio 1.34).

The route of young female no. 51 is significantly different from the previous ones and looks “anomalous”: soon after the start of migration (on the third day), having flown over the area of the Kursk magnetic anomaly, it abruptly changed its flight direction from southwest to north without any apparent reason. Then the female several times changed flight directions, gradually returning to the southwestern direction. This tracker stopped working in the south of Poland, and the route was the least efficient (2135 and 1439 km, ratio 1.48).

Coordinates of daytime shelters were obtained for all tagged bat individuals during migration for each day. During the flight, both females more than once stayed in one place for more than one day (up to 3 days). Adult female no. 46 stopped most frequently during migration (flights occurred only 54% of the nights of the entire migration), but also had the longest flights on migration nights (an average of 250 km per day). Male no. 53 made migration flights every night and had only one stop for one day at the end of the recorded route. After this, the tracker failed in data transmition. We have no information about whether the male either continued its migratory flight, or completed its migration in the area of the last track point.

As a result of our research, for the first time we obtained data on seasonal migration of the greater noctule on a daily basis over a fairly long period.

The hibernation of greater noctule apparently takes place in European countries with a Mediterranean climate, where they were met in winter [11, 12]. The general direction of the autumn migration of all three greater noctules with GPS-GSM trackers was oriented to southwest from their summer habitats in Russia to southern European countries. It corresponds to the known data for European bats and is consistent with the general direction of long-distance migrations of bats, identified on the basis of numerous returns of ringed animals, including a closely related species, the common noctule [3].

The autumn migration of the greater noctule occurs in September, but its start depends on weather conditions. Our tagged animals began migration on September 19 (in 2020) and September 28 (in 2021).

Greater noctule bats tagged in Russia crossed several countries during migration flight. Our greater noctule's GPS-GSM trackers stopped working in different European countries. Female no. 51 tracker stopped data transmission in the south of Poland on October 4, 2020. It crossed Belarus and Lithuania after Russia. Female no. 46 tracker stopped working in the east of Bulgaria on October 11, 2021. It flew over the territory of Ukraine, Moldova and Romania. Male no. 53 tracker stopped working in northwestern Italy, approximately 100 km from the French border on October 16, 2021. It crossed Ukraine, Poland, the Czech Republic, Austria and Slovenia.

The migration of greater noctules took place at night with stops during the day, which sometimes lasted more than a day. Female no. 51 made flights on September 19–26, 28–29 and October 1–2. It stop days were September 27, 30 and October 3. Female no. 46 made flights on September 28–30 and October 1−3, 7. Stop days were on October 4–6 and 8–10. Male no. 53 made flights on September 28–30 and October 1−14. It stop days were October 15. Stops lasting more than a day were apparently related to weather conditions: during rain and strong winds greater noctules don’t leave their shelters. We recorded similar behavior before migration start too.

Animals migration route is largely determined by the orography of the territory over which they fly. Adult female no. 46 flew to the hibernation site along the most optimal route, close to a straight line (ratio 1.05, 12 days between the start and finish points). The route of young male no. 53 was less optimal (ratio 1.34). It had a longer flight (19 days from the day of launch until the loss of the signal from the tracker near the potential wintering site). Medium-altitude mountains (from 1000 m and above) became an obstacle for him. After unsuccessful attempts to cross higher mountains (about 2000 m high), he changed his migration trajectory. Male no. 53 spent some time and energy flying along the northern tip of the Central European mountains (Carpathians, Sudetes) in an attempt to overcome them. At the end he was forced to cross the Alps mountains, which are about 2000 m high.

Among all obtained bat migration tracks two are normal (no. 46 and no. 53) and one anomalous: young female no. 51 flight trajectory is radically different from the other ones. Its track is S-shaped and deviates greatly from the optimal trajectory (ratio 1.48). As a result, with a total track length of 2135 km, the straight-line distance between the starting and ending points was only 1439 km. In our opinion, the migration trajectory of this animal is energetically wasteful. We did not find any obvious reasons of such anomaly flight—large orographic obstacles, forest zone boundary changes, large rivers directions, etc. The only spatial coincidence is the Kursk Magnetic Anomaly (KMA). Female no. 51 changed optimal migration direction from southwestern and flew northwards during crossing it.We assume that the reason for such track anomaly is function disruption of young female’s magnetic compass system after flying over the KMA and its restoration a few days later, when the animal gradually returned from the northern direction of flight to the southwestern. It is known that bats can use a magnetic field for orientation like birds [13]. Birds can use several compass systems for orientation, and the magnetic compass system is only one of them [14]. “Calibration” of compass systems and the ability to choose the “right” one in each specific set of conditions may depend on the life experience and individual characteristics of individuals.

Our hypothesis raises the question—why the KMA did not affect flight direction another two greater noctules that crossed it. It is possible that trajectory differences in KMA crossing were due to its spatial heterogeneity [10, 15], or individual variability in animal response to KMA magnetic field anomalies. Even in our small three animals sampling, the most optimal, close to direct, flight was made by an adult female, minimizing its energy costs.Hibernating sites of greater noctule in the climatic conditions of Poland, where the tracker of female no. 51 stopped signal transmition, are unknown. We do not exclude the possibility that it continued the journey in search of more favorable wintering climatic conditions to the south (Bulgaria and Italy), like the other two individuals.

As a result of studying greater noctule bats migration with GPS-GSM trackers several flight distance records for bats were set. The maximum distance covered by a greater noctule per day was 445 km, which is comparable to the size of some countries. This distance exceeds the known daily flights of other bat species [7], specifically the hoary bat [16], African and Australian fruit bats [5, 6]. In addition, the previous record for bat migration distance, held by the common pipistrelle bat *Pipistrellus nathusii* [17] was exceeded by 30 km: greater noctule male no. 53 covered a distance of 2515 km in a straight line (the total length of the track was 3360 km). At the same time, we do not know the subsequent path of this male and we cannot exclude that the final distance of its migration may have been even greater.

Another aspect that our data on greater noctule migration allows us to discuss is the assumption about the magnitude of genetic variability in this species. The extensive range of the greater noctule is mosaic and consists of many disparate micropopulations [18], which suggests the presence of genetic differences among them. Our results suggest that intensive genetic exchange between these micropopulations may exists: individuals from the remote northeastern part of the range can carry out long migration, flying through central European countries to southern Europe, and can make their genetic contribution to these micropopulations. Mating in *Nyctalus lasiopterus* apparently occurs in the autumn, as in its related species, *N. noctula*, the common noctule [19]. A study of common noctule genetic variability showed that the European population of this species, despite its extensive range stretching across Europe for several thousand kilometers, is genetically relatively monomorphic [20]. Further research will test our assumption and will be able to clarify whether spatially separated greater noctule populations differ from each other in genetic characteristics.
