# Erratum to: **Autumn Migration of Greater Noctule Bat (*Nyctalus Lasiopterus*): through Countries and over Mountains to a New Migration Flight Record in Bats**

**DOI:** 10.1134/S0012496623050125

**Published:** 2024-01-25

**Authors:** D. A. Vasenkov, N. S. Vasiliev, N. V. Sidorchuk, V. V. Rozhnov

**Affiliations:** 1 Severtsov Institute of Ecology and Evolution, Russian Academy of Sciences, Moscow, Russia; 2 Moscow State University, Moscow, Russia

The article “Autumn Migration of Greater Noctule Bat (*Nyctalus Lasiopterus*): through Countries and over Mountains to a New Migration Flight Record in Bats,” written by D.A. Vasenkov, N.S. Vasiliev, N.V.  Sidorchuk, and V.V. Rozhnov, was originally published Online First in Springer-Link on November 11, 2023 without Open Access. After publication, the authors decided to make the article an Open Access publication. Therefore, the copyright of the article has been changed to © The Author(s) 2023 and the article is forthwith distributed under the terms of a Creative Commons Attribution 4.0 International License (http://creativecommons.org/licenses/by/4.0/, CC BY), which permits use, duplication, adaptation, distribution and reproduction of a work in any medium or format, as long as you cite the original author(s) and publication source, provide a link to the Creative Commons license, and indicate if changes were made.

